# Advances in Cellulose-Based Hydrogels: Current Trends and Challenges

**DOI:** 10.3390/gels10120842

**Published:** 2024-12-20

**Authors:** Bogdan-Marian Tofanica, Aleksandra Mikhailidi, Costel Samuil, Ovidiu C. Ungureanu, Maria E. Fortună, Elena Ungureanu

**Affiliations:** 1 “Gheorghe Asachi” Technical University of Iasi, 73 Prof. Dr. Docent D. Mangeron Boulevard, 700050 Iasi, Romania; b.m.tofanica@gmail.com; 2 IF2000 Academic Foundation, 73 Prof. Dr. Docent D. Mangeron Boulevard, 700050 Iasi, Romania; amikhailidi@gmail.com; 3 “Ion Ionescu de la Brad” Iasi University of Life Sciences, 3 Mihail Sadoveanu Alley, 700490 Iasi, Romania; 4 Faculty of Medicine,“Vasile Goldis” Western University of Arad, 94 the Boulevard of the Revolution, 310025 Arad, Romania; 5 “Petru Poni” Institute of Macromolecular Chemistry, 41A Grigore Ghica Voda Alley, 700487 Iasi, Romania

**Keywords:** cellulose, cellulose-based gels, hydrogels, gels

## Abstract

This paper provides a solid foundation for understanding the synthesis, properties, and applications of cellulose-based gels. It effectively showcases the potential of these gels in diverse applications, particularly in biomedicine, and highlights key synthesis methods and properties. However, to push the field forward, future research should address the gaps in understanding the environmental impact, mechanical stability, and scalability of cellulose-based gels, while also considering how to overcome barriers to their industrial use. This will ultimately allow for the realization of cellulose-based gels in large-scale, sustainable applications.

## 1. The Foundation of Cellulose-Based Gels

For millennia, long before its chemical nature was understood, humans unknowingly relied on cellulose as a fundamental resource. Biomass was a source of energy, wood was used for construction, and plant fibers were used for making clothes. Since the industrialization era, cellulose’s versatility made it indispensable in traditional industries, particularly as a key component in paper production and textile manufacturing. Over time, its applications expanded, paving the way for innovations in material science and engineering. But as science progressed, researchers began to recognize its potential beyond these traditional uses. Its abundance, biodegradability, and non-toxicity made it an ideal candidate for innovative applications, including the development of gel-like substances. The transformation of cellulose into gels is not a simple process; it requires sophisticated techniques and a deep understanding of the chemistry and physics of cellulose’s polymeric nature.

**Figure 1:**
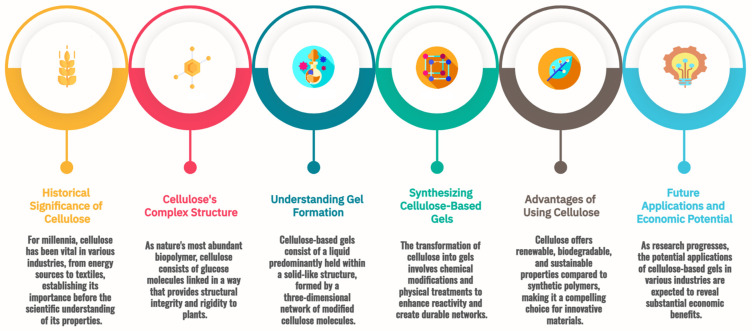
Key knowledge in cellulose-based gel research.

The journey into the world of cellulose-based gels begins with an examination of the fundamental properties of cellulose itself, as depicted in Figure 1. At first glance, it seems a humble substance, the primary component of plant cell walls, a ubiquitous presence in nature in the plant kingdom. Yet, it holds a secret—a complexity that has fascinated scientists for generations and revolutionized industries from medicine to food processing.

Cellulose, a linear polymer composed of glucose molecules, is nature’s most abundant biopolymer. Its intricate structure, formed by beta-D-glucose units linked through 1,4-glycosidic bonds, creates a rigid, insoluble network [1]. This feature makes cellulose an essential structural component in plants, contributing to their strength and rigidity.

Gels are fascinating materials because they exhibit properties of both solids and liquids. They are considered a type of colloid in which a liquid is dispersed in a solid matrix [2]. This unique structure gives gels their characteristic strength and ability to hold their shape like a solid, while also allowing them to flow and deform like a liquid under certain conditions. Essentially, gels are somewhere in between a solid and a liquid, making them incredibly versatile for various applications [3]. In the case of cellulose-based gels, this network is created by the cellulose molecules themselves, which must undergo a transformation to achieve the desired gel-like consistency [4,5,6].

The process of synthesizing cellulose-based gels often begins with modifying the cellulose fibers to make them more reactive, thus enabling them to form stronger, more durable networks. This can be achieved by introducing chemical groups to the cellulose backbone or by using physical methods like solvent exchange, heat, and pressure. These treatments open up new avenues for cellulose to interact with solvents and other chemicals, allowing it to form the intricate, web-like structures that define gels [7].

But why cellulose? Why not use other materials? The answer lies in the unique characteristics of cellulose. Unlike synthetic polymers, which are derived from petrochemicals, cellulose is renewable, biodegradable, and widely available. It offers a sustainable alternative for industries looking to reduce their reliance on non-renewable resources. Consequently, these properties render cellulose challenging to employ, particularly when the objective is to create the soft, flexible gels that will be the subject of this brief overview. This turns cellulose-based gels into a promising material for a plethora of applications.

It is important to note that this review primarily focuses on applications of cellulose-based gels that have reached TRL (Technology Readiness Level) 5 or higher. These applications have demonstrated significant potential and have been proven at the laboratory scale, indicating their readiness for further development and potential commercialization. By focusing on these advanced applications, we aim to provide a practical and scientifically robust overview of the current state of cellulose-based gel technology.

As we continue to investigate the synthesis, applications, and potential of cellulose-based gels in the forthcoming years, we will observe how these materials are deployed in real-world scenarios, thereby further substantiating the substantial economic promise of this natural material.

## 2. From Plants to Cellulose-Based Gels

The synthesis of these gels typically involves modifying cellulose in order to achieve a gel-like structure, with the process varying depending on the desired properties and application. The synthesis methods can be generally classified into physical, chemical, and combined physico-chemical approaches [8]. The final desired gel’s characteristics—such as viscosity, texture, and stability—are determined by the precise control of these factors. Different synthesis methods can yield gels with a wide range of properties, allowing scientists to tailor the material for specific applications.

### 2.1. Physical Crosslinking

Physical crosslinking is one of the most commonly used methods for cellulose gel synthesis. In this approach, the cellulose chains are organized into a gel network by non-covalent interactions such as hydrogen bonding, van der Waals forces, or hydrophobic interactions. These interactions allow the cellulose molecules to form a three-dimensional network, resulting in gelation [9].

One typical method of physical crosslinking is the dissolution of cellulose in an appropriate solvent, followed by the gradual addition of water or another non-solvent, which causes the cellulose chains to interact and form a gel [10]. This approach avoids the need for chemical reagents, making it a more environmentally friendly and biocompatible method. The rheological properties and gel strength can be controlled by adjusting the solvent composition, temperature, and concentration of cellulose [11].

### 2.2. Chemical Crosslinking

Chemical crosslinking involves the use of chemical agents to covalently bond cellulose molecules, which enhances the mechanical stability and structural integrity of the gel. This approach typically requires the cellulose to first be chemically modified by introducing functional groups that can react with crosslinking agents [12]. Common crosslinking agents used for cellulose include glutaraldehyde, epichlorohydrin, or poly-carboxylic acids [13].

In this method, cellulose is usually dissolved in a solvent, and a crosslinking agent is added to form covalent bonds between the hydroxyl groups of cellulose molecules, resulting in the formation of a gel network [14]. The degree of crosslinking can be controlled by adjusting the concentration of the crosslinking agent, reaction time, and temperature. Chemical crosslinking can significantly enhance the mechanical properties and stability of the gel, making it suitable for applications requiring high strength and durability [15].

### 2.3. Hybrid Approaches

Hybrid methods combine physical and chemical techniques to achieve the desired properties of cellulose-based gels. For example, cellulose can first be physically cross-linked to form an initial network, and then chemical crosslinking can be employed to further stabilize the gel structure [16]. Alternatively, cellulose can be combined with other polymers or nanoparticles to enhance its properties, such as improving mechanical strength or introducing new functionalities like antibacterial properties [17].

One approach involves the combination of cellulose with natural polysaccharides, like chitosan or alginate, to form structurally integrated gels [18]. These gels can offer a balance of the mechanical properties of cellulose with the bioactive potential of other natural polymers, making them ideal for use in biomedical and environmental applications.

Another common method of synthesizing cellulose-based gels involves solvent exchange. In this process, cellulose is first dissolved in a solvent, such as an ionic liquid or a strong alkali solution, and then the solvent is gradually exchanged with a non-solvent, such as water or alcohol [19]. The change in solvent composition leads to the formation of a gel network. The gelation process is often driven by the solubility of cellulose in the solvent and subsequent precipitation as the solvent is replaced by a non-solvent.

This technique is often used when the goal is to preserve the natural structure of cellulose while forming a gel. It is particularly useful for applications where biocompatibility is important, such as in wound dressings or drug delivery systems.

## 3. The Unique Properties of Cellulose-Based Gels

Cellulose-based gels, with their remarkable properties, stand as a testament to the ingenuity of science and the boundless potential of nature’s resources. From their unique molecular structure to their impressive ability to absorb water, these gels offer a wealth of possibilities that extend across diverse industries. Their biocompatibility, mechanical strength, and environmental sustainability make them a valuable alternative to synthetic materials, paving the way for a future where innovation and sustainability go hand in hand [20].

### 3.1. Structural Integrity: A Network of Strength

The key to understanding cellulose-based gels lies in their molecular structure. As previously mentioned, cellulose is a polymer made up of long chains of glucose molecules. When cellulose undergoes the process of gelation, these chains form a dense, cross-linked network. This network is responsible for the gel’s ability to retain water while maintaining its form [21]. The degree of crosslinking and the arrangement of cellulose fibers can vary, which leads to significant differences in the gel’s behavior [16].

At the core of these gels’ structural integrity is the combination of cellulose’s crystalline and amorphous regions. In its natural state, cellulose is highly crystalline, meaning its molecules are tightly packed and organized in a regular pattern [22]. However, when it is dissolved and reformed into a gel, the cellulose molecules may become partially disordered. This disorder, combined with the crystalline regions, allows the gel to maintain a balance between strength and flexibility [23]. The extent of this balance can be tailored by adjusting the concentration of cellulose and the gelation conditions.

The result is a gel that exhibits impressive mechanical properties, such as resistance to deformation and the ability to hold its shape under pressure. These characteristics make cellulose-based gels suitable for applications where stability and structural integrity are essential, such as drug delivery systems, wound dressings, and even as a matrix for 3D printing [24].

### 3.2. Hydrophilicity: The Ability to Absorb and Retain Water

One of the most striking properties of cellulose-based gels is their ability to absorb and retain water. This hydrophilic nature is a direct result of the hydroxyl groups (-OH) present on the glucose molecules that make up the cellulose chains [25]. These groups form hydrogen bonds with water molecules or other hydroxyl groups, creating a highly interactive environment within the gel [26]. This interaction allows cellulose gels to hold a significant amount of water within their structure, resulting in a material that appears solid but is, in fact, mostly water [27].

This high water retention capacity makes cellulose-based gels ideal for a variety of applications, particularly in the medical and cosmetic industries. For example, in wound care, these gels can provide a moist environment that accelerates healing by preventing the dehydration of the wound site [28]. Similarly, in cosmetic formulations, the water-binding ability of cellulose-based gels makes them valuable in hydrating products like moisturizers and serums [29].

The water retention capability also plays a crucial role in the texture and consistency of foods [30]. Cellulose-based gels are often used in the food industry as thickeners or stabilizers, where their water-holding properties help to maintain the desired viscosity and texture [31]. This property also allows for the creation of low-calorie, water-rich food products, making cellulose-based gels a popular choice in the growing market for healthy and sustainable food options [32].

### 3.3. Biocompatibility: A Natural Fit for Medical Applications

One of the most compelling reasons to use cellulose as a base for gel materials is its inherent biocompatibility. As a naturally occurring polymer, cellulose does not trigger adverse immune responses in humans, making it ideal for applications that come into direct contact with the body [33]. This property has led to a surge in the use of cellulose-based gels in the medical field, particularly for wound care, drug delivery, and tissue engineering [34].

When applied to wounds, cellulose-based gels create a barrier that prevents bacterial invasion while promoting moisture retention. This creates an optimal healing environment, reducing the risk of infection and speeding up recovery [35]. Additionally, the gels are biodegradable, meaning that they break down naturally over time, eliminating the need for removal and reducing the risk of complications [36].

In drug delivery systems, cellulose-based gels can be engineered to release active pharmaceutical ingredients in a controlled and sustained manner. The gel matrix can encapsulate drugs, allowing them to be gradually released into the body over time, improving treatment outcomes and minimizing side effects. The biocompatibility of cellulose ensures that these systems are safe for long-term use [37].

### 3.4. Mechanical Properties: Flexibility and Strength

Another key feature of cellulose-based gels is their unique balance of flexibility and strength. The mechanical properties of the gel are determined by factors such as the degree of crosslinking, the molecular weight of the cellulose, and the gelation conditions [38]. In general, cellulose-based gels exhibit a high level of flexibility, allowing them to conform to different shapes and structures without cracking or breaking [39].

This flexibility is particularly beneficial in medical applications, where gels may need to be applied to irregularly shaped wounds or to delicate areas of the body [40]. The gel’s ability to stretch and adapt without losing its integrity makes it a versatile material in this context. Moreover, the ability to adjust the stiffness of the gel through the synthesis process provides additional flexibility for use in a wide range of applications.

On the other hand, cellulose-based gels are also strong enough to withstand mechanical stresses. The cross-linked network within the gel imparts strength, allowing it to maintain its structure under pressure or when subjected to external forces. This strength, combined with its flexibility, makes cellulose-based gels ideal for applications where durability and resilience are essential [41].

### 3.5. Tunable Rheology: Control over Texture and Viscosity

Rheology—the study of the flow and deformation of materials—is another important consideration when discussing the properties of cellulose-based gels. The texture and viscosity of a gel are critical factors in determining its suitability for different applications [42]. Cellulose-based gels offer an exceptional degree of tunability in this regard, as their rheological properties can be easily adjusted during synthesis [43].

By altering factors such as the concentration of cellulose, the type of solvent used, the degree of crosslinking, and the temperature at which gelation occurs, it is possible to create gels with a wide range of viscosities, ranging from thin and pourable to thick and spreadable. This flexibility in rheology makes cellulose-based gels highly adaptable to the specific needs of various industries [44].

In food production, for instance, the ability to fine-tune the viscosity of cellulose-based gels allows manufacturers to create products with the desired mouthfeel and consistency [45]. Similarly, in pharmaceuticals, controlled rheology can be used to design gels that flow easily for topical application or maintain their shape when used as injectable carriers for drugs [46].

### 3.6. Environmental Sustainability: A Green Alternative

Perhaps the most significant property of cellulose-based gels is their environmental sustainability [47]. Unlike synthetic polymers derived from petroleum, cellulose is a natural, renewable resource. It is biodegradable and non-toxic, meaning that it poses minimal environmental impact when disposed of. This makes cellulose-based gels an attractive option for industries seeking to reduce their carbon footprint and adopt more sustainable practices [48].

In industries such as agriculture, cellulose-based gels can be used in biodegradable films for packaging or as water-retaining agents in soil, helping to conserve water and promote sustainable farming practices [49]. Moreover, their ability to absorb pollutants and toxins has made them an appealing material for environmental cleanup applications, such as oil spill remediation [50].

As concerns over plastic waste and environmental degradation continue to grow, the demand for sustainable alternatives like cellulose-based gels will only grow, further driving the development of these materials for a wide range of applications [51].

## 4. The Applications of Cellulose-Based Gels

Cellulose-based gels are gaining widespread recognition due to their natural origin, biodegradability, versatility, non-toxicity, water retention, and so on. Their applications span numerous industries, ranging from healthcare and food production to environmental remediation and material science. In the current work, we have intentionally focused on applications that are at TRL 5 or higher—validated technologies in relevant environments, out of the laboratory scale, as these are closer to practical implementation and commercialization. The ability to modify cellulose gels to suit specific needs—whether through the introduction of functional groups or by combining them with other natural polymers—continues to expand their use, offering sustainable and efficient alternatives to traditional materials. Some examples of cellulose-based gels used in different fields are presented in Table 1.

### 4.1. Medical and Pharmaceutical Applications

The medical field has seen some of the most profound transformations thanks to cellulose-based gels. Their versatility, biocompatibility, and water-holding properties make them an invaluable resource in various medical applications, particularly in wound care and drug delivery [60].

Cellulose-based gels are increasingly used in wound care products, particularly in the treatment of burns, chronic wounds, and diabetic ulcers [35,61,62]. These gels form a moist, protective barrier over the wound, preventing dehydration and promoting faster healing. The ability to retain large amounts of water makes these gels ideal for keeping the wound site hydrated, reducing pain, and accelerating tissue regeneration [63]. The biodegradability of cellulose also ensures that there is no need for removal, unlike traditional synthetic bandages that may require medical intervention [64].

In some advanced dressings, cellulose-based gels are incorporated with antimicrobial agents or growth factors to further enhance healing and reduce the risk of infection [65]. The gels can be tailored to provide specific levels of hydration and firmness, adapting to the needs of different types of wounds. Their ability to form a conformal dressing that molds to the contours of the wound makes them particularly effective at treating irregularly shaped injuries [66].

Another key application of cellulose-based gels is in controlled-release drug delivery systems. The gels serve as matrices that can encapsulate pharmaceutical compounds and release them over an extended period. This controlled release minimizes the need for frequent dosing, improving patient compliance and ensuring a steady therapeutic effect [67]. For example, in the treatment of chronic conditions such as diabetes or pain management, cellulose gels can be used to deliver drugs like antibiotics, insulin, or opioids in a manner that ensures consistent, sustained release [68,69].

In some cases, cellulose gels are used in ocular drug delivery systems, where they help to keep medications in contact with the eye surface for longer periods [70]. Additionally, cellulose-based gels are being explored in targeted drug delivery, where the gel’s properties can be fine-tuned to release drugs at specific sites within the body, such as tumors or infected tissues, enhancing the precision and effectiveness of treatment [71]. 

### 4.2. Food and Beverage Industry

In the food industry, cellulose-based gels have become essential ingredients for improving texture, stability, and shelf-life. These gels are often used as thickeners, stabilizers, emulsifiers, and gelling agents in a variety of food products, providing an alternative to synthetic or animal-derived ingredients [31,32].

Cellulose-based gels are widely used to modify the viscosity of food products. In sauces, soups, dressings, and dairy products, cellulose gels help to achieve the desired consistency without altering the flavor or introducing unhealthy fats [72]. Their ability to bind water efficiently allows for the creation of smooth, creamy textures that appeal to consumers while reducing the need for additives or preservatives.

In addition to thickening, cellulose-based gels act as stabilizers in emulsions, where they help to maintain the uniform dispersion of oil and water phases in products like mayonnaise, ice cream, and salad dressings [73]. This helps to prevent separation and ensures the longevity of the product, even under fluctuating temperatures.

As the demand for healthier food options increases, cellulose-based gels offer an exciting opportunity for fat and calorie reduction. By using cellulose gels as fat replacers, food manufacturers can reduce the caloric content of products while maintaining the desired mouthfeel and texture. This is particularly useful in low-fat or reduced-calorie versions of processed foods, where maintaining sensory qualities such as creaminess or thickness is crucial for consumer acceptance [74].

Cellulose-based gels also play a role in the encapsulation of nutrients and functional ingredients, such as vitamins, minerals, and probiotics [75]. By encapsulating these ingredients in a cellulose matrix, manufacturers can protect them from degradation during processing, improve bioavailability, and ensure that nutrients are released at the optimal time during digestion. This application is especially important for functional foods that promote health and wellness.

### 4.3. Cosmetics and Personal Care

The beauty and personal care industry has also embraced the unique properties of cellulose-based gels. From moisturizers to anti-aging creams, these gels provide a natural, effective way to deliver hydration, texture, and stability in a wide range of cosmetic formulations.

Cellulose-based gels are commonly found in hydrating skin-care products like moisturizers, face masks, and serums. Thanks to their water retention capabilities, these gels help to deliver intense hydration to the skin, leaving it feeling soft and smooth. They also form a breathable layer on the skin that prevents water loss while maintaining a natural barrier, which is particularly useful for dry or sensitive skin [76,77].

In addition to moisturizing, cellulose-based gels are used in anti-aging treatments due to their ability to enhance the delivery of active ingredients [78]. They act as carriers for anti-aging compounds such as peptides, antioxidants, and hyaluronic acid, helping these ingredients to penetrate the skin more effectively. The gentle, non-irritating nature of cellulose-based gels makes them ideal for formulations designed for delicate skin around the eyes or for sensitive individuals [79].

Cellulose-based gels are also valued for their ability to stabilize emulsions, ensuring that ingredients like oils and water-based compounds stay evenly mixed in cosmetic products [80]. This prevents separation and ensures that the product maintains its consistency throughout its shelf life.

Their use in gels for hair styling, for example, allows for easy application while providing hold and texture without the greasy or heavy feel of traditional waxes or creams.

### 4.4. Environmental Applications

Beyond healthcare and consumer goods, cellulose-based gels are proving to be valuable in environmental applications, particularly in waste management and pollution control.

Cellulose-based gels have been studied for their potential for absorbing and removing oil from water, making them effective agents in oil spill cleanups. Their high water-holding capacity and ability to form strong, cohesive networks allow them to trap oils and other hydrophobic substances, facilitating the efficient removal of contaminants from the environment. This ability to absorb large volumes of oil while maintaining their form is what makes cellulose-based gels an attractive alternative to traditional oil spill cleanup methods, which are often less efficient and more damaging to the ecosystem [81,82].

In agriculture, cellulose-based gels are being used to improve water retention in soils, particularly in arid regions. The gels can absorb large amounts of water, releasing it gradually to the roots of plants, helping to conserve water and promote sustainable farming practices [83,84,85]. This application is especially critical in areas where water scarcity is a growing concern, as it can significantly reduce the need for irrigation while improving crop yield.

The absorbent nature of cellulose-based gels has also led to their use in wastewater treatment. These gels can trap heavy metals, toxins, and other pollutants, making them ideal for cleaning contaminated water [86,87,88,89]. In addition to their ability to absorb pollutants, cellulose-based gels are biodegradable, meaning that they do not contribute to environmental pollution after use, unlike many synthetic materials.

### 4.5. Industrial and Technological Applications

Cellulose-based gels have even begun to find a place in high-tech applications, including 3D printing, nanotechnology, and energy storage.

In the rapidly advancing field of 3D printing, cellulose-based gels are being explored as materials for creating biodegradable, sustainable prints. Their gel-like consistency allows for smooth, high-resolution printing, while their biocompatibility makes them an attractive option for applications in medicine and biotechnology [90]. Researchers are currently investigating ways to incorporate cellulose-based gels into printable bio-inks for creating tissues or scaffolds for regenerative medicine [91].

The unique properties of cellulose-based gels have also found applications in energy storage systems, such as batteries and supercapacitors [92,93]. Researchers are exploring the use of cellulose-based gels as electrolyte materials, where they help to improve conductivity and efficiency while offering a more sustainable, biodegradable alternative to synthetic electrolytes [94]. This could represent a significant step forward in the development of eco-friendly energy storage solutions.

## 5. Innovation and Intellectual Property: Startups and Patents

Cellulose-based gels are no longer confined to the pages of scientific journals or laboratory experiments. They have emerged as a versatile and sustainable class of materials, with applications across diverse industries, including pharmaceuticals, food, cosmetics, and environmental technology [95].

Derived from cellulose, the most abundant biopolymer on Earth, these gels offer a distinctive combination of properties, including biocompatibility, biodegradability, and adjustable mechanical strength. Their remarkable properties have allowed them to make a significant impact across a wide range of industries, ranging from healthcare to food processing and even environmental conservation.

This has stimulated considerable interest in their development and commercialization, as evidenced by the growing number of patents and startups.

### 5.1. Intellectual Property in Cellulose-Based Gel Technologies

Patents are of great importance in the protection of the intellectual property associated with innovations in cellulose-based gels, the fostering of technological advancement, and the enabling of economic opportunities. The development of these materials frequently involves the use of novel chemical modifications, crosslinking techniques, or specific formulations that are tailored to meet the demands of specialized applications. As a result, patent documents provide a rich resource for the understanding of the state of the art in this field, as well as for the identification of key players and trends.

Cellulose-based materials are widely used across various industries, with numerous research groups working to improve existing technologies for their production and modification. This is reflected in the large number of patents granted for cellulose materials. For instance, as many as 8053 patents on cellulose hydrogels were published between 1965 and 2021 [96]. A detailed analysis showed that patents were granted for the application in food, paper, textile, packaging, and pharmaceutical industries. In biomedical contexts, the hydrogels may serve as antibacterial agents; plays roles in tissue regeneration, wound dressings, and transdermal patches; and serve as biosorbents for cartilage tissue engineering. While [96] primarily focuses on classifications such as countries, patent holders, and patent families, it offers limited insight into the technological aspects of the patents. Reference [95], on the other hand, examines the patents granted for hydrogels based on all kinds of natural polymers, with limited examples on cellulose. To build upon these findings, we will explore several additional examples.

Table 2 presents patents on hydrogels from non-derivatized cellulose. The majority of these patents focus on physical gelation and hybrid methods. Physical gelation involves the use of solvents (e.g., NaOH, DMAc/LiCl, NNMO) to dissolve cellulose, followed by gelation (the formation of new hydrogen bonds between the macromolecules of cellulose) and regeneration. The hybrid approach combines physical gelation with the addition of chemical crosslinking agents (e.g., epichlorohydrin, glutaraldehyde, or citric acid), which enhances composite properties such as absorbency and mechanical strength.

An interesting approach, described in [97], utilizes a separately prepared superabsorbent hydrogel (a mixture of acrylamide and sodium polyacrylate). The hydrogel powder is dissolved in water and added to cellulose pulp during papermaking. Interaction between the hydroxyl groups of cellulose and the functional groups (carboxyl and amide) of the hydrogel significantly improves the composite properties.

As the primary cellulose source, the studies predominantly used plant-based cellulose (bleached and unbleached sulfate cellulose, wood pulp, cotton cellulose) and nanocellulose (NFC, NCC, and bacterial cellulose). The use of nanocellulose demonstrates promising results. For instance, producing hydrogels from NFC [98] does not require any specialized methods or solvents, as it happens after direct mixing with water. This characteristic makes NFC-based materials highly favorable. However, the mechanical strength of such hydrogels remains a concern.

Applications of hydrogels derived from non-derivatized cellulose include the following fields:

- Medicine: wound dressings, drug delivery systems, contact lenses, and corneal bandages;
- Environmental protection: water purification and pollutant adsorption;
- Cosmetics: moisturizing gels and cooling patches;
- Household and hygiene products: diapers, sanitary pads, and highly absorbent materials.

The abundance of patents related to cellulose-based hydrogels demonstrates the significant interest of researchers and industries in this material. A recent patent analysis revealed active innovation in the field, with patents primarily focused on medicinal applications, including prostheses and coatings, emphasizing the functional and physical properties of cellulose derivatives, such as macromolecular gels and hydrogels.

**Table 2:** Patents on hydrogels based on non-derivatized cellulose.

Cellulose Type	Method of Preparation	Details of the Method	Application	Year	Country	Reference(s)
Cellulose nano-fibers (CNF) derived from softwood or hardwood pulp prepared by TEMPO oxidation and high-pressure homogenization	Hybrid approach (chemical crosslinking combined with physical gelation)	The CNF dispersion (2–3 wt%) is mixed with sodium silicate and sulfuric acid as a curing agent. The mixture is stirred, allowed to stand, and aged at 65–80 °C for 6–12 h to achieve a hydrogel with 75–90 wt% water content.	Anisotropic conductive materials, medical devices, pollutant adsorption, food gels	2004	China	[99]
Microcrystalline cellulose (MCC) from biodegraded softwood and industrial hemp hurds	Physical crosslinking	MCC is dissolved in a DMAc/LiCl solvent system using a temperature cycle (heating to 55–58 °C for 5 min, cooling to 18–20 °C, and repeating). Gelation at room temperature for 3–5 days. Solvent is removed by washing with distilled water, and the hydrogel is dried via ambient conditions or lyophilization.	Biomedical and pharmaceutical applications, including wound dressings, drug delivery systems, and tissue engineering	2024	Russia	[100]
Cellulose nanofibrils (NFCs)	Physical crosslinking	The hydrogel is formed by dispersing NFC in water, creating a physically cross-linked network without the use of chemical crosslinking agents.	Medical applications, including wound care products, due to its biocompatibility and ability to maintain a moist environment conducive to healing	2024	European Union	[98]
Bleached sulfate softwood pulp, bleached sulfate hardwood pulp, and thermomechanical bleached wood pulp	Hybrid approach (combines papermaking technology with the addition of preformed hydrogel particles into the cellulose matrix)	The superabsorbent hydrogel is prepared separately from acrylamide and sodium poly-acrylate; then, it is dispersed in water to form a 1–10% (wt.) solution. The hydrogel suspension is mixed for 10–15 min to limit swelling and then added to the cellulose pulp. Interaction between the hydroxyl groups of cellulose and the functional groups (carboxyl and amide) of the hydrogel enhances composite properties.	Composite material for highly absorbent sanitary and hygienic products, such as diapers, pads, and sheets	2023	Russia	[101]
Pineapple peel residue cellulose	Hybrid approach (cold plasma-initiated chemical crosslinking combined with physical gelation)	Dissolving cellulose in NaOH/urea, adding acrylic acid and a crosslinking agent, and using cold plasma to generate hydroxyl free radicals for polymerization.	Environmental remediation, particularly wastewater treatment; adsorbs heavy metal ions (Zn²⁺, Cd²⁺, Cr³⁺) with a removal rate of 56–72%	2021	China	[102]
Microcrystalline cellulose (MCC)	Hybrid approach	Dissolution of cellulose in solvents (e.g., ionic liquids, DMSO, DMF, or aqueous alkaline solutions), followed by crosslinking using agents such as epichlorohydrin, glutaraldehyde, and citric acid.	Biomedical uses (e.g., wound dressings, drug delivery systems) and environmental applications (e.g., adsorbents for water purification)	2019	International	[103]
Specific type is not specified (cellulose in general)	Hybrid approach	Dissolving cellulose in an alkaline solution (e.g., NaOH) and adding two-dimensional layered materials. Crosslinking agents are used, but not specified.	Photothermal therapy and other biomedical applica-tions	2019	China	[104]
Plant-based cellulose (e.g., wood pulp, cotton, microcrystalline cellulose) and bacterial cellulose (e.g., *Gluconacetobacter xylinus*).	Physical crosslinking	The process includes cellulose activation using a solvent, dissolution of the activated cellulose (DMAc/LiCl, CED, NMMO, NaOH/water, IL) and gelation of the solution under relative humidity conditions ranging from 30% to 80% at 35 °C. Additional drying and rehydration steps can be applied to enhance mechanical properties. The process also involves molding to produce products such as contact lenses.	Manufacturing contact lenses, including temporary corneal bandages, with high oxygen permeability, biocompatibility, and low endotoxin levels	2016	USA	[105]
Unbleached or bleached cellulose	Physical crosslinking	The process involves the depolymerization of cellulose through acid hydrolysis at high temperatures, followed by mechanical dispersion in water. The mechanical treatment uses high-shear forces to break agglomerates and disperse nanocrystals, forming a three-dimensional hydrogel structure through hydrogen bonding and physical interactions.	Composite materials, rheology modifiers in drilling fluids and cement mixtures, biodegradable polymers, paints, emulsions, and the pharmaceutical, medical, food, and cosmetic industries	2013	Russia	[97]
Bacterial cellulose	Physical crosslinking	The bacterial cellulose aquagel is immersed in a gelatin solution, forming a stable hydrogel structure. In some formulations, Borneolum Syntheticum and Mentholum are added to enhance the cooling and pain-relieving properties of the hydrogel.	Medical materials for temperature reduction and pain relief, specifically used as a reusable hydrogel plaster for fever management, particularly in infants and children, as well as for physical therapy and pain relief	2012	China	[106]
Dissolving pulp, paper pulp, cotton linters, or cotton, with DP 150–6200	Physical crosslinking	Dissolving cellulose (2–13.5% in NMMO or up to 30% in IL) and precipitating with a water-soluble salt (e.g., sodium sulfate) to regenerate a hydrogel with a cellulose II structure. The method allows optional incorporation of functional substances for targeted uses.	Medical and cosmetic applications (wound dressings, cooling agents, patches, scaffolds for regenerative medicine), plant substrates in agriculture	2009	Austria	[107]
Plant-derived cellulose (e.g., wood pulp, flax, straw, cotton) and bacterial cellulose (*Gluconacetobacter xylinus*); composites of bacterial and plant cellulose are also mentioned	Physical crosslinking	Activating cellulose using solvents such as DMAc/LiCl, methanol, ethanol, or water. The activated cellulose is dissolved, and the solution is gelled under controlled humidity (30–80%) and temperature (35 °C). Optional processes include drying, rehydration, and molding into medical devices.	Medical purposes, including wound-healing materials, drug delivery systems, contact lenses, corneal bandages, and other medical devices	2009	Germany	[108]
Viscose (sodium cellulose xanthate solution)	Physical crosslinking	Cellulose is dissolved in an aqueous or organic solvent system (e.g., viscose, DMSO/paraformaldehyde, or DMAc/LiCl). Coagulation and regeneration by immersing the cellulose solution in an aqueous solution containing 30–90 wt% organic solvents such as methanol, ethanol, acetone, or tetrahydrofuran.	Ophthalmic materials (soft contact lenses, artificial corneas, and vitreous body replacements) in cosmetics (base for gels and creams) and filtration membranes (ultrafiltration and dialysis systems)	1999	USA	[109]

### 5.2. Startup Landscape: Bridging Research and Market

Technological startups contribute to the development of the entrepreneurial ecosystem, the introduction of innovations, the creation of new jobs, and accelerated economic growth, especially in high-tech industries. They stimulate competition by implementing new technologies and business models, encouraging established companies to evolve. The activities of startups help to address complex social and economic challenges, laying the foundation for a more sustainable future [110]. The contribution of technological entrepreneurship is particularly evident in fast-growing sectors such as biotechnology, where innovations drive the transformation of key economic industries.

According to forecasts, by 2030, the biotechnology market, including nanobiotechnology, is expected to demonstrate steady growth, reaching USD 3.88 trillion with a compound annual growth rate (CAGR) of 13.96% [111]. During this period, technological entrepreneurship will focus on implementing solutions based on artificial intelligence and robotic technologies, as well as on the development of medical innovations, advanced materials for healthcare, and the improvement of agricultural practices, including biotechnological solutions to increase yields, enhance resistance to pests and diseases, and reduce the carbon footprint [111,112,113]. Strengthening partnerships between the private and public sectors for the successful implementation of these technologies will become a critical task for the future.

Cellulose, as a renewable polymer, has become the foundation for creating a substantial number of startups. According to the international online platform Venture Radar, there are more than 250 such companies. Firms in Europe, Asia, and other regions are actively exploring new ways to use cellulose to create sustainable materials, including hydrogels. These materials find applications in medicine, agriculture, environmental protection, cosmetics, and other industries. Examples of such startups include FineCell (Bromma, Sweden), Anziboo (Nairobi, Kenya), and ANPOLY (Pohang-si, Republic of Korea), which are already integrating their technologies into commercial practice (Table 3).

Table 3 provides data on startups developing cellulose hydrogels. Several startups have successfully attracted significant investments for scaling technologies, building pilot plants and establishing industrial facilities. For instance, in 2023, FineCell secured EUR 1 million for constructing a pilot plant, while ANPOLY raised USD 3.73 million over several years. Regional differences in the investment climate play a notable role. Europe and Asia remain the most favorable regions for such startups. For example, companies in Jena, Germany (JeNaCell) and Helsinki, Finland (UPM Biomedicals) have successfully attracted substantial investments and established strategic partnerships, ensuring long-term support for their projects.

The diversity of hydrogel applications is also noteworthy, as they are used in various industries. These applications range from medical solutions, such as wound dressings developed by JeNaCell, to agricultural developments, including hydrogels developed by Anziboo and Sumatrix Biotech designed to improve soil structure and fertility. This highlights the versatility of cellulose materials and their importance in different sectors of the economy.

Thus, startups in the field of cellulose hydrogels, while relatively small in number, are actively advancing due to the unique properties of cellulose, its availability, and support from investors. Companies from Sweden, Germany, and South Korea stand out in particular, underscoring the importance of regional investment strategies for the development of such projects.

Most startups specializing in cellulose hydrogels have emerged in the past five years, though some have longer histories (e.g., JeNaCell, founded in 2012). Recent news from these companies indicates that most are actively growing, providing an optimistic outlook for the future.

## 6. Perspectives on the Future of Cellulose-Based Gels

**Figure 2:**
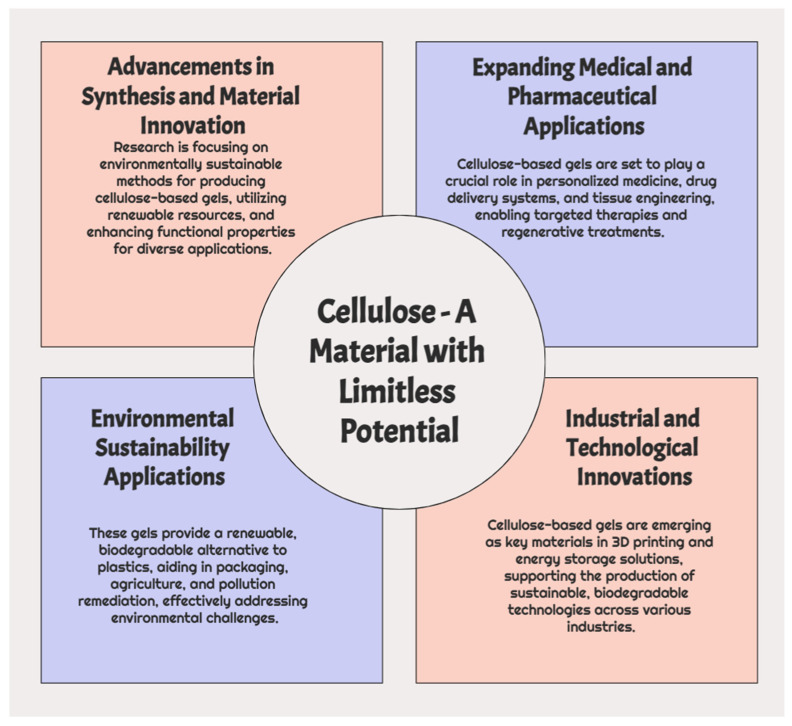
Advancing cellulose-based gels: future perspectives.

The potential of cellulose-based gels becomes ever more exciting as we progress. These materials are poised to play an increasingly significant role in addressing some of the most pressing challenges in fields ranging from healthcare to environmental sustainability. With their natural abundance, biodegradability, and extraordinary versatility, cellulose-based gels are not just a temporary solution but a long-term answer to some of the modern world’s most pressing issues, as depicted in Figure 2.

Looking toward the future, the field of cellulose-based gels is positioned for significant advancements [114]. Researchers are striving to enhance the properties of these gels, with the objective of making them stronger, more versatile, and more cost-effective [115]. The continued development of novel solvents and crosslinking agents will facilitate the creation of cellulose-based gels with enhanced, more specialized properties, thereby opening up new frontiers in science and technology for the next generation of wearable devices.

Advances in wearable epidermal sensors have revolutionized the way that physiological signals are captured and measured for monitoring health [116]. Cellulose-based gels, with their tunable optical properties—modifiable by factors such as temperature, pH, light, and electric fields—offer promising opportunities for integration into wearable epidermal sensors. In addition, the integration of machine learning techniques into wearable sensors and bioelectronics has further transformed real-time sensing and data analysis for personalized healthcare [117]. This is in addition to their traditional applications, which remain essential and continue to be of significant value [118]. Supervised and unsupervised learning algorithms can uncover complex patterns and relationships within large, high-dimensional datasets, providing clinical-grade insights.

This synergy between cellulose-based gels and cutting-edge scientific research domains could pave the way for advances in the fundamental understanding of these materials, leading to more accurate, reliable, and innovative applications in various fields.

### 6.1. Advances in Synthesis and Material Innovation

One of the most promising areas of research and development for cellulose-based gels is in the field of their synthesis. Although cellulose has been known and utilized for centuries, the recent advances in its processing and gelation are truly groundbreaking.

In light of the growing global emphasis on sustainability, there is a heightened focus on the development of cellulose-based gels produced through more environmentally conscious and sustainable methods. Current synthesis methods frequently necessitate the utilization of solvents and chemicals that can prove detrimental to the environment or result in significant production costs. However, researchers are developing more environmentally friendly processes that utilize water or non-toxic solvents to create cellulosic gels. This transition towards green chemistry in the production of cellulose-based materials has the potential to markedly reduce the environmental impact of their manufacture, thereby enhancing their appeal for broader utilization.

Moreover, scientists are investigating the potential of utilizing cellulose derived from unconventional sources, such as agricultural waste, bacterial, or algae, for the production of cellulose gels. These sources are renewable and abundant, offering a sustainable alternative to cellulose derived from traditional wood sources. As the technology for extracting and processing cellulose improves, it is likely that an even wider range of natural sources will be utilized, thereby reducing the strain on forests and other traditional resource industries.

Another promising avenue of research is the functionalization of cellulose-based gels. By incorporating a variety of chemical groups or additives into the cellulose matrix, researchers can modify the gels to exhibit specific properties that enhance their performance in particular applications. To illustrate, cellulose-based gels can be modified to enhance their mechanical strength, thermal stability, or responsiveness to environmental stimuli, such as temperature or pH.

The functionalization of cellulose gels may facilitate their use in drug delivery systems, whereby the gels release active ingredients in response to changes in body temperature or pH. This could offer a more controlled and efficient means of therapy. Similarly, the development of cellulose-based gels with enhanced antibacterial or antifungal properties could facilitate the creation of new medical applications, including those related to wound care and implants.

### 6.2. Expanding Medical and Pharmaceutical Applications

The utilization of cellulose-based gels in the field of medicine is already well established; however, future developments promise to make these materials even more vital in healthcare. As new applications emerge, cellulose gels are likely to become a fundamental component of advanced medical treatments.

In light of the increasing emphasis on personalized medicine, whereby treatments are tailored to the specific needs of individual patients, cellulose-based gels may prove to be a crucial component in the effective delivery of drugs and therapies. By modifying the gel’s characteristics, it is feasible to develop bespoke drug delivery systems that release active ingredients at specified doses and times in accordance with the patient’s distinctive condition. To illustrate, a cellulose gel could be designed to deliver a specific drug in response to particular biomarkers present in an individual’s blood, thus offering a targeted approach to treatment with minimal side effects.

Cellulose-based gels have the potential to revolutionize the field of regenerative medicine, particularly in the domain of tissue engineering. As research progresses, it is conceivable that cellulose-based gels could serve as scaffolds for the growth of new tissues or organs. These gels have the capacity to mimic the extracellular matrix of natural tissues, thereby providing support for cell growth and differentiation. By incorporating cells and growth factors into these gels, researchers could develop more effective treatments for injuries, burns, and degenerative diseases. The ability to create biodegradable scaffolds that eventually dissolve into harmless by-products adds an element of safety to these approaches, thereby enhancing their suitability for medical applications.

Due to their capacity to provide a moist healing environment and to deliver active agents directly to the site of an injury, cellulose-based gels will continue to evolve for use in wound care, particularly for chronic conditions that are difficult to treat. The continued development of gels that release bioactive molecules, such as growth factors or anti-inflammatory drugs, may facilitate enhanced healing rates for patients with conditions such as diabetes, where impaired wound healing is a common occurrence. Furthermore, the integration of regenerative elements into cellulose-based gels may facilitate the development of innovative treatments for patients with severe or non-healing wounds.

### 6.3. Environmental Sustainability Applications

The future growth of cellulose-based gels is likely to be driven by the compelling argument for sustainability. In the context of the global challenges of climate change, resource depletion, and plastic pollution, cellulose represents a renewable and biodegradable alternative to synthetic materials. The continued development of cellulose-based gels is inextricably linked to a broader transition towards sustainable and environmentally friendly materials across all industrial sectors.

One of the most pressing environmental issues of our time is the accumulation of plastic waste, and cellulose-based gels present a promising solution. In contrast to petroleum-based plastics, cellulose gels are biodegradable and can decompose naturally without releasing deleterious chemicals into the environment. As the global focus on reducing plastic consumption intensifies, cellulose-based gels may replace plastic in a variety of applications, including packaging, disposable products, and even single-use medical devices.

The utilization of cellulose gels in the field of packaging may prove to be a particularly revolutionary development. In addition to their biodegradable nature, these gels can be produced using renewable resources, thereby reducing the environmental impact of packaging waste. The potential for cellulose-based gels to be used in food packaging is also being investigated. The ability of these gels to retain moisture could assist in the preservation of food and the reduction in food waste.

In light of the growing pressure on water resources brought about by climate change, the utilization of cellulose-based gels in agricultural settings represents a promising avenue for the conservation of this vital resource. These gels have the capacity to absorb and retain considerable quantities of water, subsequently releasing it to plants in arid environments where irrigation is constrained. As agricultural practices increasingly incorporate sustainability measures, cellulose-based gels could assist farmers in reducing their reliance on water-intensive irrigation, thereby contributing to global water conservation efforts.

Furthermore, cellulose-based gels can be utilized in the remediation of environmental contamination, where their absorptive characteristics can facilitate the removal of pollutants from soil or water. The gels can be formulated to selectively absorb specific toxins, such as heavy metals or hydrocarbons, thereby providing a natural and biodegradable solution to pollution.

### 6.4. Industrial and Technological Innovations

Cellulose-based gels are not only beneficial for environmental and medical applications; they are also likely to be significant in technological and industrial developments.

In the field of three-dimensional printing, cellulose-based gels are rapidly emerging as a novel and innovative material. Their biocompatibility, ease of processing, and versatile properties render them optimal candidates for bio-inks utilized in bioprinting. As researchers continue to refine cellulose-based bio-inks, they may play a significant role in the creation of living tissues, organs, and prosthetics, representing a leap forward in regenerative medicine and personalized healthcare.

Furthermore, cellulose-based gels have the potential to be employed in the production of eco-friendly 3D-printed items for a diverse range of industries, including packaging and automotive components. As the demand for sustainable, biodegradable materials increases, cellulose gels may prove to be a crucial solution for reducing the environmental impact of 3D printing processes.

The necessity for environmentally friendly energy storage solutions is driving the advancement of cellulose-based materials for use in devices such as batteries and supercapacitors. These materials have the potential to facilitate the development of more efficient and eco-friendly energy storage systems, which are vital for the future of renewable energy infrastructure. The incorporation of cellulose-based gels into energy storage systems may enable the creation of lighter, more sustainable devices that enhance the performances of electric vehicles, solar panels, and other renewable energy technologies.

## 7. Conclusions: A Material with Limitless Potential

The synthesis of cellulose-based gels represents a highly versatile and rapidly evolving field of research. A variety of methods, including physical crosslinking, chemical crosslinking, and hybrid approaches, permit the tailoring of gel properties to align with the requirements of particular applications. Although cellulose gels offer a number of advantages, including biodegradability, biocompatibility, and sustainability, further research is required to enhance their scalability, mechanical stability, and processability in order to facilitate their use in large-scale industrial applications.

A deeper comprehension of the nuances of synthesis methodologies and their impact on the ultimate characteristics of the gels will facilitate the advancement of future innovations in this domain.

Cellulose-based gels, with their remarkable properties, serve as a testament to the ingenuity of scientific inquiry and the boundless potential of natural resources. From their distinctive molecular structure to their remarkable capacity to absorb water, these gels offer a plethora of potential applications across a range of industries. Their biocompatibility, mechanical strength, and environmental sustainability render them a valuable alternative to synthetic materials, thereby paving the way for a future in which innovation and sustainability are mutually reinforcing.

The potential applications of cellulose-based gels are as diverse as they are transformative. From their revolutionary role in healthcare to their impact on the food industry, cosmetics, and the environment, these gels offer a sustainable, versatile alternative to many conventional materials. As research into their properties and applications continues to evolve, it is possible to envisage a future in which these gels will be employed in a multitude of new and innovative ways.

The future of cellulose-based gels presents a plethora of promising prospects and untapped potential. Due to their distinctive characteristics, sustainability, and adaptability, these materials are well placed to transform a multitude of industries, spanning healthcare, food, technology, and environmental conservation. As developments in material science continue, it seems reasonable to anticipate that cellulose-based gels will become involved in a growing number of pioneering applications. This could lead to the development of sustainable responses to some of the world’s most pressing challenges.

As researchers and innovators continue to advance the frontiers of cellulose gel technology, one thing is clear: the future holds great promise for these remarkable materials. The ongoing exploration and development of cellulose-based gels are not only expanding their practical applications but also contributing to a more sustainable and technologically advanced world.

## Figures and Tables

**Table 1:** Cellulose-based gels, examples, and their use in various industries.

Cellulose-Based Gels	Description	Applications	References
Carboxymethyl Cellulose (CMC) Gel	CMC is a water-soluble cellulose derivative commonly used to form gels. It is produced by chemically modifying cellulose with carboxymethyl groups, making it highly effective as a thickening and gelling agent.	○ Pharmaceuticals: CMC is used in controlled-release drug delivery systems, where it helps release drugs slowly over time. ○ Food Industry: It is often found in processed foods, such as ice cream, salad dressings, and sauces, where it serves as a stabilizer and thickener. ○ Cosmetics: CMC is also used in personal care products like lotions and shampoos to improve texture and consistency.	[52]
Hydroxypropyl Cellulose (HPC) Gel	HPC is another cellulose derivative, created by introducing hydroxypropyl groups into the cellulose structure. HPC forms a gel when mixed with water, which is stable and transparent.	○ Pharmaceuticals: HPC gels are used in the production of tablets and controlled-release formulations. ○ Personal Care: They are widely used in the formulation of hair care products and cosmetics, providing texture and enhancing product performance. ○ Food: HPC can be used in gluten-free baking to improve dough consistency	[53]
Methylcellulose (MC) Gel	MC is a cellulose derivative where the hydroxyl groups are methylated. This modification allows methylcellulose to form gels when heated, which is reversible (the gel dissolves when cooled).	○ Food Industry: MC is used as a fat replacer in low-calorie foods and as a thickener in sauces, soups, and desserts. ○ Pharmaceuticals: It is used in tablet formulations to control the release of drugs. ○ Cosmetics: In personal care products, it is used for its gelling properties in lotions, creams, and hair care products.	[54]
Cellulose Acetate (CA) Gel	CA is a derivative of cellulose where the hydroxyl groups are replaced with acetate groups. When dissolved in certain solvents, it can form gels with desirable properties like film formation and controlled release.	○ Pharmaceuticals: CA gels are used in drug delivery systems, particularly in controlled- or sustained-release formulations. ○ Cosmetics: It is used in cosmetics and personal care products, including skin creams and hair gels, due to its gel-forming ability and smooth texture.	[55]
Cellulose Ester Gel	Cellulose esters are cellulose derivatives in which ester groups replace the hydroxyl groups. These gels have unique properties such as biodegradability, and they are used in various industries.	○ Pharmaceuticals: It is used as controlled-release agents in drug delivery systems. ○ Coatings and Paints: In the manufacturing of coatings, cellulose ester gels provide good film-forming properties.	[56]
Cellulose Nanocrystal (CNC) Gel	CNCs are nanoscale particles derived from cellulose fibers. These particles can be dispersed in water to form a gel-like consistency with high mechanical strength and excellent stability.	○ Biomedical: CNC gels are being explored for drug delivery systems, where they can encapsulate drugs and control their release. ○ Materials Science: CNCs are used in the development of eco-friendly composites and films, replacing petroleum-based plastics. ○ Environmental: CNC gels have potential applications in water purification and oil spill cleanup due to their high absorbent properties.	[57]
Alginated Cellulose Gel	This gel combines cellulose and alginate, two naturally occurring polysaccharides, to create a unique material. The combination of these two substances produces gels with good stability and high moisture retention.	○ Wound Care: Alginated Cellulose gels are often used in advanced wound care products because of their ability to create a moist healing environment and promote faster healing. ○ Pharmaceuticals: It is sed in controlled-release drug delivery, where it helps release active ingredients over time. ○ Food Industry: Alginate Cellulose gels are used in food applications, where they serve as texturizing agents.	[58]
Cellulose-Based Hydrogel for Agriculture	These hydrogels are formed by the crosslinking of cellulose with various other agents to create a gel that retains water and slowly releases it to plants.	○ Agriculture: Cellulose-based hydrogels are used for soil moisture retention, reducing the need for frequent irrigation in drought-prone areas. This helps to conserve water while improving crop yields.	[49]
Cellulose-based Gel for Oil Spill Remediation	Some cellulose-based gels are formulated specifically to absorb oils and other hydrophobic substances	○ Environmental Cleanup: These gels are highly effective at absorbing oils from water, making them invaluable in oil spill cleanup efforts. Their biodegradable nature makes them a more sustainable option than synthetic absorbents.	[59]

**Table 3:** An overview of startups focused on cellulose hydrogels.

Company Name	Country	Hydrogel Production Technology	Key Product Features	Business Model	Investments	Latest News	Source
ANPOLY	South Korea	Hydrogel production from TEMPO-oxidized cellulose nanofibrils (T-CNF).	A CNF diameter less than 10 nm, with a high length-to-diameter ratio (~1:100), providing excellent water dispersibility. The hydrogel is transparent, odorless, and highly viscous.	Development and production of nanocellulose materials for various industries, including biomedicine and cosmetics, aiming to replace synthetic polymers with eco-friendly alternatives.	USD 3.73 million received in 10 funding rounds; the latest round took place in April 2024. In January 2021, the company was valued at USD 16 million. Revenue for 2020 amounted to USD 65,610.	In November 2023, ANPOLY received the CES 2024 Innovation Award in the category “Sustainability, Eco-Design, and Smart Energy” for its Re:ancel™ T-CNF product.	en.anpolyinc.com/
Anziboo	South Africa	I-kuze Biogel hydrogel is made from bamboo powder. Some versions include biochar.	Eco-friendliness, biodegradability, water retention up to 500 g/g, soil health improvement, the prolongation of product freshness, and controlled drug release.	The production of biohydrogel fertilizers for agriculture and medicinal patches for healthcare. Collaboration with small-scale farmers and sustainable production practices.	Information on investments is not available.	In July 2024, the launch of I-kuze Biogel sales was announced, with pre-orders becoming possible.	www.anziboo.com/
Bios Hydrogel	Italy	The base component is cellulose from paper waste, wood, rice husk, straw, and seaweed. The synthesis process avoids aggressive chemicals.	Water retention, soil structure improvement, biodegradability, and increased soil fertility.	The sale of sustainable solutions for agriculture and vertical farming.	Founded in 2019, an application has since been submitted for university spin-off status.	Victory at the BioInItaly Investment Forum (2020); in June 2021, a technology for improving agricultural soil use was presented.	www.bioshydrogel.com
FineCellOx	Sweden	The energy-efficient processing of wood MFC and NC cellulose (CellOx) into hydrogels.	Transparency, biodegradability, and versatility (binding agent).	Replacing petrochemicals in personal care products, cosmetics, paints, thin-film coatings, and 3D bioprinting. Partnerships with brands.	EUR 1 million investment (2023), pilot plant launch (2024), and industrial production (2027).	In March 2024, FineCell was recognized by leading beauty industry publications for its innovative approaches and products.	https://finecell.se/
JeNaCell	Germany	The biosynthesis of cellulose using *Komagataeibacter xylinus* bacteria; cellulose with ~100 nm fibers.	Soft, flexible, moist material; used in wound dressings (e.g., epicite^®^) for treating burns and chronic wounds.	Production of biosynthetic cellulose for medical devices and dermatology; part of Evonik since 2021.	Investments from Evonik Venture Capital in 2015; full acquisition by Evonik in 2021.	In 2021, the epicite^®^ balance wound dressing for chronic wounds was launched.	www.jenacell.com
Scion	New Zealand	Nanocellulose for hydrogel production is extracted from seaweed using residual materials from agriculture.	High strength, lightness, and biodegradability	Areas: Biomedicine, packaging, and composite material production. Partnership with AgriSea for industrial-scale nanocellulose hydrogel production.	1.5 million New Zealand dollars, including a loan of 750,000 dollars from the New Zealand Ministry of Economic and Regional Development.	In August 2022, the creation of the world’s first commercial facility for producing nanocellulose from seaweed was announced.	www.scionresearch.com/
Sumatrix Biotech	Turkey	The fermentation of industrial waste containing glucose and nitrogen to produce nanostructured biocellulose.	A superhydrophilic, biocompatible hydrogel with high water retention capacity used in vertical farming and hydroponics.	The production of biomaterials for agriculture, medicine, and the textile industry.	Supported by accelerators and incubators.	In 2023, an alternative to leather made from fermented waste was introduced; in 2024, products for hydroponic farming were developed.	en.sumatrixbiotech.com/
UPM Biomedicals	Finland	FibGel™ is made from nanofibrillated cellulose derived from birch wood. The hydrogel is developed and manufactured according to medical devices quality standards.	Biocompatibility, tunable properties, stability, and non-degradability. The material is designed for long-term use in the human body without triggering immune reactions or forming fibrotic capsules.	Tissue restoration, orthopedics, aesthetics, drug delivery, cell transplantation; collaboration with medical device developers; clinical trials with partners in 2025.	Information on investments is not available.	On 24 October 2024, UPM Biomedicals announced the launch of FibGel™, the world’s first injectable nanocellulose hydrogel for medical devices.	www.upmbiomedicals.com/
		GrowDex^®^-A consists of nanocellulose derived from birch wood, modified with avidin for binding biotinylated molecules.	Imitation of the extracellular matrix to support cell growth and differentiation; transparency and improved imaging quality during microscopy.	Sale of hydrogel for 3D cell cultures and biomedical research; collaboration with research organizations and commercial partners.	Information on investments is not available.	On 29 September 2021, UPM Biomedicals announced a partnership with PerkinElmer Health Sciences, Inc., to provide solutions for high-throughput 3D cell screening in early-stage drug development.	

This review is not intended for promotional purposes, and the authors are in no way affiliated with any of the listed startups. All information presented here was sourced from publicly available sources, including websites and news articles dedicated to the listed startups. All websites have been accessed during the preparation of the manuscript, during December 2024.

## Data Availability

No new data were created or analyzed in this study.
